# A whole-body FDG PET/MR atlas for multiparametric voxel-based analysis

**DOI:** 10.1038/s41598-019-42613-z

**Published:** 2019-04-16

**Authors:** Therese Sjöholm, Simon Ekström, Robin Strand, Håkan Ahlström, Lars Lind, Filip Malmberg, Joel Kullberg

**Affiliations:** 1 Department of Surgical Sciences, Uppsala University, Uppsala, Sweden; 2 Department of Information Technology, Uppsala University, Uppsala, Sweden; 3 Antaros Medical AB, Mölndal, Sweden; 4 Department of Medical Sciences, Uppsala University, Uppsala, Sweden

## Abstract

Quantitative multiparametric imaging is a potential key application for Positron Emission Tomography/Magnetic Resonance (PET/MR) hybrid imaging. To enable objective and automatic voxel-based multiparametric analysis in whole-body applications, the purpose of this study was to develop a multimodality whole-body atlas of functional 18F-fluorodeoxyglucose (FDG) PET and anatomical fat-water MR data of adults. Image registration was used to transform PET/MR images of healthy control subjects into male and female reference spaces, producing a fat-water MR, local tissue volume and FDG PET whole-body normal atlas consisting of 12 male (66.6 ± 6.3 years) and 15 female (69.5 ± 3.6 years) subjects. Manual segmentations of tissues and organs in the male and female reference spaces confirmed that the atlas contained adequate physiological and anatomical values. The atlas was applied in two anomaly detection tasks as proof of concept. The first task automatically detected anomalies in two subjects with suspected malignant disease using FDG data. The second task successfully detected abnormal liver fat infiltration in one subject using fat fraction data.

## Introduction

With the advent of integrated Positron Emission Tomography/Magnetic Resonance (PET/MR) systems, it is now possible to acquire whole-body functional PET data co-registered with morphological and functional MR data. This has great potential in systemic diseases such as oncological^1^, metabolic^2^ and cardiovascular diseases where the spatially and temporally aligned whole-body PET/MR multiparametric images could offer advantages in detection tasks, when following diseases over time and in therapy response assessments.

Quantitative multiparametric imaging has been highlighted as a potential key application for PET/MR^3^. However, multiparametric datasets are extremely information rich, making visual assessment both challenging and time-consuming. Quantitative analysis tends to involve heavy data reduction, using a few a priori specified measurements of interest, commonly with manually or semi-automatically defined regions of interest^4–6^. These approaches are both time-consuming and subject to intra- and inter-observer variability. However, in an attempt to utilise the full potential of multiparametric datasets, new workflows for large-scale analysis are emerging^5,7^. Common features of these approaches are the aims for a more detailed quantitative data analysis and automation, and hence a reduction in user bias.

Voxel-wise statistical analysis of registered (spatially normalised) data has been extensively reported for brain imaging studies, with the most commonly used analysis framework being Statistical Parametric Mapping (SPM)^8^. A number of standardised PET and MR brain templates of healthy controls have been developed within SPM, allowing for registration of subjects to the respective template and objective statistical inferences^9–12^. Rigid or affine transformations are most commonly employed in brain imaging applications for which the inter-subject anatomical variability is small compared to body applications. The larger inter-individual variability seen for body applications makes image registration challenging and more complex non-rigid transformations are needed. Image registration of region-specific body applications, followed by voxel-wise analysis, has been reported^13,14^. However, healthy control templates similar to the SPM approach have in general not been used. Whole-body registration methods in humans have mainly relied on atlas based approaches for the purpose of e.g. PET attenuation correction^15^ and muscle volume quantification^16^. In this study we use Imiomics, a framework for whole-body registration for the purpose of voxel-wise statistical analysis^7^. In Imiomics, whole-body fat-water MR images are registered using a three-step pipeline, allowing for voxel-wise analysis and statistical inferences following registration. Usage of inherently co-registered PET/MR data gives the further opportunity for automated and quantitative multiparametric PET and MR analysis using the Imiomics workflow.

The purpose of this work was to develop a multimodality whole-body atlas of functional 18F-fluorodeoxyglucose (FDG) PET and anatomical fat-water MR data of adults for the purpose of multiparametric voxel-based analysis. The term atlas is used as it both provides a template for registration and contains parametric maps of voxel-wise normal values for each modality. As a proof of concept, a secondary purpose of the study was to perform automated voxel-wise anomaly detection using the atlas.

## Methods

### Subjects and Imaging Protocol

In this prospective study, 89 healthy volunteers were included (43 males, 46 females, mean age 68.4 years, age range 51.0–76.7 years). The 89 subjects constituted the subset of the Swedish Epihealth study^17^ that had undergone PET/MR imaging, with imaging performed between August 2015 and November 2017. The study was approved by the Uppsala Ethics Committee and conducted in accordance with the Declaration of Helsinki. Written informed consent was obtained from all participants.

Whole-body FDG PET and MR Attenuation Correction (MRAC) data were simultaneously acquired on an integrated 3.0 T PET/MR scanner (Signa PET/MR, GE Healthcare). To ensure a blood glucose level below 8 mmol/l, subjects fasted a minimum of 6 hours prior to injection with 2 MBq/kg of FDG. Whole-body PET/MR imaging started 113 ± 8 min post injection of FDG and consisted of a 3 min/bed PET scan and a Dixon MRAC scan (TE 1.67 ms, TR 4.05 ms, flip angle 5°, voxel size 2.0 × 2.0 × 2.6 mm). Standard PET reconstruction was performed using a 3D Time-of-Flight iterative reconstruction (VUE Point FX, 2 iterations, 28 subsets, standard 5 mm filter, voxel size 3.1 × 3.1 × 3.1 mm) with attenuation correction based on the MRAC scan. Ten stations per subject were acquired and then assembled on the scanner platform to form whole-body fat and water MR, and PET datasets. Fat and water fraction images were computed using the MRAC data^18^.

**Figure 1:**
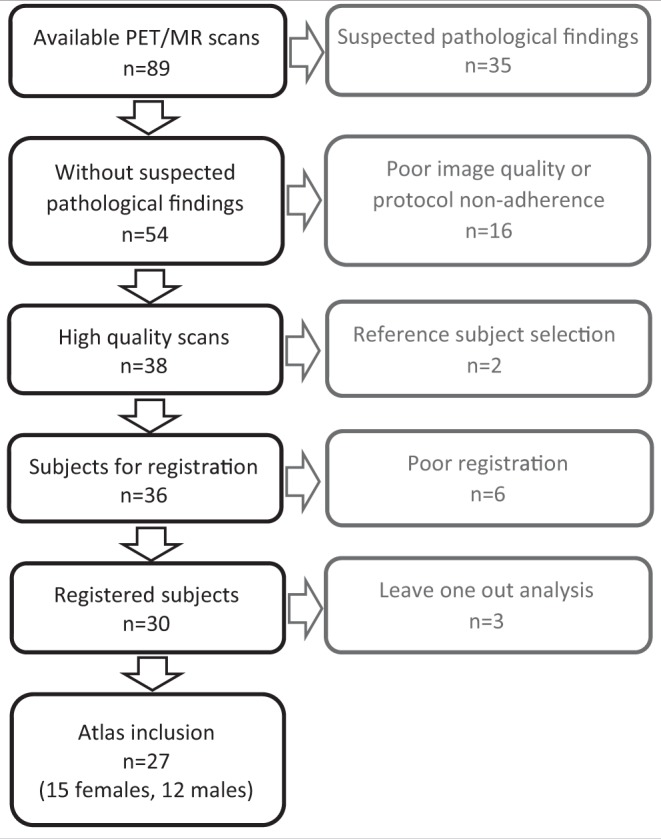
Flowchart of subject inclusion (black) and exclusion (grey).

Conservative atlas inclusion criteria were used to ensure that all atlas entries represented high quality scans with normal physiological values (see flow chart in Fig. 1). Each PET/MR scan was first assessed for suspected pathological findings by a Radiologist experienced in PET and MR imaging reporting (operator 1, H.A., with 20 years or operator 2 with 10 years of experience). Subjects with suspected pathological findings (n = 35) were excluded (Table 1). Image quality and protocol adherence were also assessed. From this step, a further 16 subjects were excluded: as the imaging data included metal artefacts (n = 10), the subject moved between bed positions (n = 1), the whole-body was not within the field of view (n = 2), the PET scan start had been delayed (n = 3), the subject had not been fasting (n = 1) and as problems occurred in the MR image reconstruction (n = 1). Note that the total number of excluded subjects was 16, but that an overlap exists between exclusion criteria.

**Table 1:** Suspected pathological findings leading to exclusion of subjects from the atlas.

Finding	Modality	n
Suspected malignant tumour disease	PET + MR	14
Degenerative changes	PET	6
Morphological brain changes	MR	4
Suspected benign tumour disease	MR	3
Muscle inflammation	PET	2
Lung changes	MR	2
Sarcoidosis	PET	1
Trochanteritis	PET	1
Fracture	MR	1
Tooth infection	PET	1

### Multiparametric atlas

**Figure 2:**
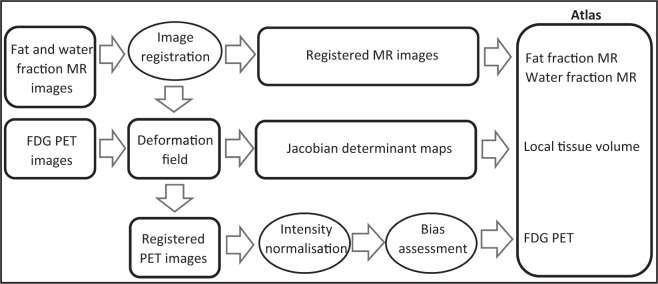
Flowchart showing input and output image data (squares) and major processes (circles) involved when creating the multiparametric atlas.

The steps involved when creating the multiparametric atlas are shown in Fig. 2.

#### Image Registration

Image registration was performed to transform all imaging data from native space into a common reference space. Two representative subjects were selected as reference spaces, one male and one female (Table 2). The selection was based on Body Mass Index (BMI), with the reference subjects having a BMI close to the mean BMI of their respective group, and visual assessment of the subjects’ positioning in the scanner and fat-water distribution.

**Table 2:** Characteristics of the reference subjects and subjects included in the atlas. Data is presented as mean (SD).

	Male		Female	
	Atlas	Reference	Atlas	Reference
n	12	1	15	1
Age (years)	67.1 (6.1)	67.2	69.6 (3.5)	67.9
Height (cm)	177.1 (8.2)	175	163.1 (4.5)	168
Weight (kg)	75.5 (9.6)	68	59.7 (7.3)	62
BMI (kg/m^2^)	24.0 (2.4)	22.2	22.4 (2.0)	22.0
Fat mass* (%)	22.5 (4.1)	29.0	30.7 (5.5)	30.7
Glucose (mmol/l)	4.9 (0.6)	5.6	4.9 (0.3)	3.9
Cholesterol (mmol/l)	5.1 (1.3)	6.2	6.1 (1.3)	5.9
Systolic blood pressure (mmHg)	132.2 (16.1)	144	127.6 (22.0)	118
Smoking** (n)	0	0	0	0
Diabetic (n)	0	0	0	0
Medication, high blood pressure (n)	3	0	0	0
Hospitalised, stroke (n)	0	0	0	0
Hospitalised, heart attack (n)	1	0	0	0
FDG injection (MBq)	159.6 (17.5)	142.0	125.1 (14.3)	128.1
PET scan time p.i. (min)	111.2 (2.4)	111.6	112.4 (5.1)	111.2

*Percentage fat of total weight.

**Currently smoking.

Whole-body fat and water fraction MR images were registered to the male or female reference space using the Imiomics pipeline as described by Strand *et al*.^7^ with modification. Imiomics provides a three-step pipeline; registering first bone sections, then water fraction images and lastly fat fraction images. The intent of the bone registration is to find a reasonable initial displacement of large bone segments. Displaced bone segments are then assumed to be correctly aligned, hence locked in place by applying these as hard constraints in the subsequent registration stages. These hard constraints restrain subsequent stages from further displacement of bones. The parametric method used for the water and fat fraction registration stages was in this study replaced by a non-parametric method^19^. The non-parametric method allows for a true voxel-to-voxel mapping providing better accuracy compared to the parametric method, which relies on a parameterisation of data points. As with the parametric approach, the regularisation was set to restrict the deformation and avoid unrealistic deformations. A slightly stricter regularisation was set for the water fraction images compared to the fat fraction images with the assumption that the deformation of muscle tissue is more homogeneous than adipose tissue.

The arm positioning was not standardised in this study and there were cases where the arms spanned outside the field of view. For this reason, the arms were removed from the images by using a semi-automatic method that cut the arm by the humeral head. As the PET and MR images were inherently co-registered, the deformation fields obtained from the MR registration were also used to deform the corresponding FDG PET data to the relevant reference space.

Visual evaluation was performed for all registered PET and MR data. Subjects with poor registration results were excluded (n = 6, Fig. 1). Errors were noted to arise due to problems when registering parts of the abdomen (n = 3), from non-standard subject positioning (n = 2) and small metal artefacts (n = 1).

#### PET Intensity Normalisation

To account for inter-subject variability in e.g. FDG injection, scan time post injection etc., intensity normalisation was performed for all registered PET data using the mediastinal blood pool as the reference tissue^20^. Volumes of Interest (VOIs) were manually delineated over the aortic arch on the MR water fraction images of the reference subjects and used to automatically obtain the mediastinal blood pool mean FDG uptake for all registered data. Intensity normalisation was then performed by voxel-wise division of each subject’s FDG data with the mean mediastinal blood pool uptake.

#### PET Bias Assessment

To ensure that the intensity of each subject’s FDG PET data contributed approximately equally to the atlas, outlier detection was performed by a leave-one-out approach. The FDG data of each subject was compared to the remaining subjects’ FDG data using a voxel-wise two-sample t-test (p < 0.001, no correction for multiple comparisons). For noise reduction, only clusters containing a minimum of 10 statistically significant voxels were considered. A body mask was used to only include within-body voxels and single subject data was smoothed using a Gaussian filter (Full Width at Half Maximum (FWHM) equals two times the voxel size). Outliers were detected by assessing the total number of statistically significant voxels for each subject, leading to the exclusion of three subjects (Fig. 1). When examining the areas of statistical significance for the outliers it proved to be due to increased leg muscle FDG uptake.

#### Atlas creation

An atlas containing parametric maps of fat and water fraction MR, local tissue volume and FDG PET for the male and female subjects, respectively, was composed. The fat and water fraction MR and FDG PET atlas parts were created by voxel-wise computation of the mean and standard deviation (SD) of the registered and intensity normalised PET/MR data, while the local tissue volume part of the atlas was obtained by voxel-wise calculation of the mean and SD of the Jacobian determinant. The Jacobian determinant has proved a useful tool for assessing the local tissue volume post-registration^21^. It quantifies the local volume change caused by applying the deformation produced by the registration, i.e. the local volumetric difference between native and reference space. A determinant between zero and one implies local contraction while a value above one implies local expansion. In this study, the logarithm of the Jacobian determinant was computed to obtain a normal distribution of the local tissue volume. For the logarithm of the Jacobian determinant, a value less than 0 indicates compression, and a value greater than 0 indicates expansion.

#### Atlas Measurements

To facilitate comparisons with other publications and evaluate the adequacy of the atlas data representing a healthy cohort, selected tissues were segmented and the mean and SD of fat fraction, organ volume and FDG uptake were assessed for the male and female atlas constituents. All segmentations were performed manually in 3DSlicer^22^ using transaxial fat and water fraction MR atlas images in reference space. To assess FDG uptake, a medical physicist (T.S.) outlined VOIs for the cerebellum, lungs, blood pool aorta, liver, spleen, psoas muscle and gluteal adipose tissue. Volumes were drawn to not include tissue borders and major vessels. Five consecutive slices were used for the cerebellum, while ten consecutive slices were used for the remaining tissues. To enable comparison with published data, non-normalised Standardised Uptake Value (SUV) FDG data (corrected for body weight) was used for the measurements. The same VOIs were used to assess fat fraction in the liver, psoas muscle and gluteal adipose tissue. For organ volumetry a nuclear medicine physician (5 years of experience) segmented abdominal organs, including lungs, liver, spleen and kidneys. By using the Jacobian determinant data, organ volumes were obtained for each atlas subject and the mean and SD organ volumes calculated for the male and female atlas subjects.

Fat fraction and SUV were also measured in native space. For this purpose, the VOIs outlined in reference space were transformed to each patient’s native space. This was performed using the inverse of the subject-specific deformation fields obtained in the registration process. An assessment of all VOIs was then performed in 3DSlicer^22^ and VOIs were manually adjusted to not include tissue borders and major vessels. To evaluate if the registration process introduces quantitative bias in the measured values, the tissue measurements performed in native and reference space were compared using a paired t-test (p < 0.05).

### Anomaly detection

As the atlas holds voxel-wise intensity values of expected normal values for a particular tissue, imaging data of subjects with suspected pathology can statistically be compared with the atlas and non-normal regions can be detected. A possible application is anomaly detection and classification. Two anomaly detection tasks were set up as proof of concept; a tumour detection task performed on the PET data and a task identifying abnormal fat infiltration using fat fraction data.

For tumour detection one male subject (age 63 years, BMI 22.3 kg/m^2^) and one female subject (age 69 years, BMI 24.9 kg/m^2^) with suspected oesophageal cancer and lymphoma, respectively, were chosen from the Epihealth PET/MR cohort. The PET/MR imaging data of these subjects were registered and intensity normalised using the same methods and parameters as the atlas subjects. A body mask was used to only include within-body voxels in the analysis and single subject PET data was smoothed using a Gaussian filter (FWHM equals two times the voxel size).

Automatic tumour detection was performed by comparing each subject’s PET imaging data to the atlas using a voxel-wise two-sample two-sided t-test (p < 0.001, no correction for multiple comparisons)^23^. This created a probability map showing the location of statistically significant regions. The following criteria were then imposed on the detected regions (MATLAB R2018a):

i. Non-normal regions should have a diameter >10 mm^4^.
ii. Non-normal regions should have a mean FDG uptake larger than the mean liver FDG uptake^6^.

Ground truth segmentation was obtained by consensus manual outlining between a nuclear medicine physician (5 years of experience) and a radiologist (H.A.) using 3DSlicer^22^. Lesions with non-normal FDG uptake were manually outlined with lesion borders identified using the fat-water MR data in native space. For all manually outlined lesions adhering to the lesion size and FDG uptake criteria described above, maximum Standardized Uptake Value (SUV_max_) were compared with SUV_max_ of the corresponding automatically detected lesions.

In the fat detection task, a male subject (age 67 years, BMI 29.0 kg/m^2^) included in the atlas and that had an elevated liver fat infiltration (median fat percentage of 7.1) was compared to the other male atlas subjects using voxel-wise two-sample two-sided t-tests on registered fat fraction data. As the liver fat percentage was borderline abnormal, this subject was still included in the final atlas. However, the subject in question was removed from the atlas for this particular test. A probability map was constructed, outlining significant regions (p < 0.05). In contrast to the tumour detection task, no explicit classification was performed.

## Results

### Multiparametric atlas

**Figure 3:**
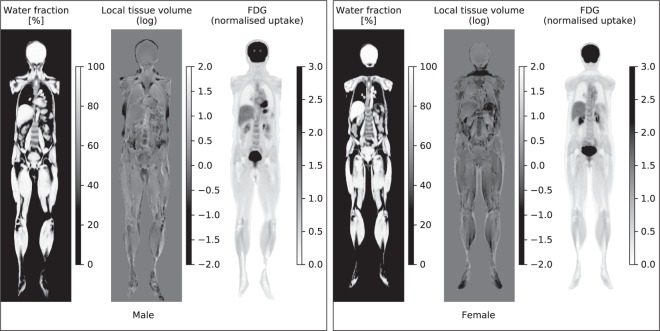
A representative coronal slice of the multiparametric atlas for the male (left) and female (right) subjects. For each gender the mean water fraction, local tissue volume and normalised FDG uptake are shown. The fat fraction is not shown as it is the inverse of the water fraction. The local tissue volume has been computed from the logarithm of the Jacobian determinant, meaning a negative value corresponds to a mean contraction and a positive value to a mean expansion when deforming the subjects to reference space.

Representative coronal parametric maps of the different parts of the atlas are shown in Fig. 3, containing data from 12 male and 15 female subjects. Characteristics for the reference and atlas subjects are summarized in Table 2 and the complete atlas is presented online as Supplemental Videos S1 and S2. Overall, organ borders are well-defined indicating good registration results. An increased variability is obtained in the feet and parts of the abdomen. These are both problematic areas for the registration procedure; feet due to non-standardised positioning in the scanner and parts of the abdomen most likely due to a varying amount of air-tissue interfaces for different subjects.

**Figure 4:**
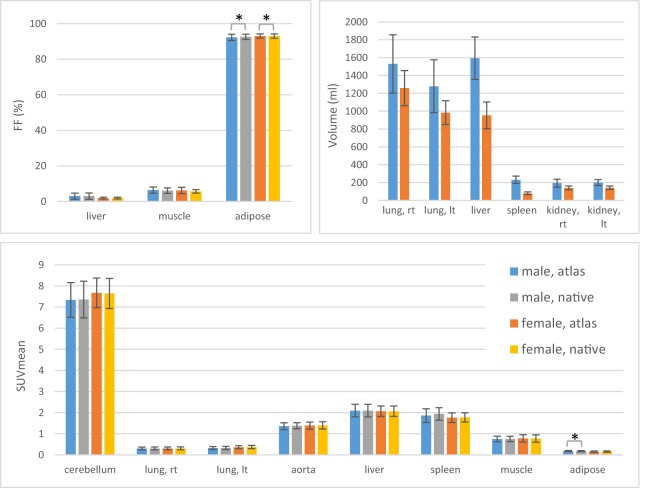
Fat fraction, organ volume and SUV_mean_ measured from manual segmentations for the male and female atlas subjects. For the fat fraction and SUV_mean_, measurements have been made in reference (atlas) and native space. The mean is shown with the standard deviation as error bars. Statistical significance is indicated with an asterisk.

Results from the normal tissue segmentation of atlas subjects in native and reference space are shown in Fig. 4. A high fat fraction is as expected measured in adipose tissue, while a relatively low fat infiltration is seen in muscle and liver. Also anticipated is the low mean Standardised Uptake Value (SUV_mean_) measured in the lungs, adipose tissue and muscle, and the higher SUV_mean_ measured in the more metabolically active cerebellum, liver and spleen. For both fat fraction and FDG uptake, similar values are seen for males and females. On the contrary, larger abdominal organ volumes were measured for males compared to females. When comparing fat fraction and SUV tissue measurements performed in native and reference space, no statistical difference was observed for the cerebellum, lungs, aorta, liver, spleen and muscle (p < 0.05). However, a quantitative bias was seen for adipose tissue, for which the male and female fat fraction and male SUV measurements showed statistically significant differences between native and reference space.

### Anomaly detection

**Figure 5:**
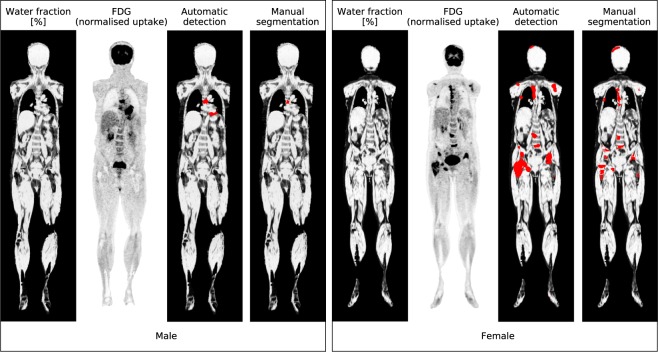
FDG anomaly detection task performed for the male (left) and female (right) subjects with suspected oesophageal cancer and lymphoma, respectively. For each subject, a representative coronal slice is shown of the registered water fraction and normalised FDG uptake images, and of the automatically detected and manually segmented tumours. Detected and segmented lesions are displayed in red and overlaid on the water fraction image. A false positive lesion can be seen for the male subject, likely due to increased motion artefacts in the heart.

A representative coronal slice of the registered water fraction and FDG imaging data, and automatically detected and manually segmented lesions for the male and female subjects are shown in Fig. 5. A total of 42 lesions were outlined manually; 38 for the female and 4 for the male. The same total number of lesions was detected automatically, but 31 for the female and 11 for the male. The total number of false positives and false negatives were 21 and 11 lesions, respectively. The false positives mainly stem from physiological uptake in the brain, bladder, heart and intestines, and the false negatives mainly stem from small nodules. One false negative however corresponded to a larger lesion in the thoracic vertebrae.

**Figure 6:**
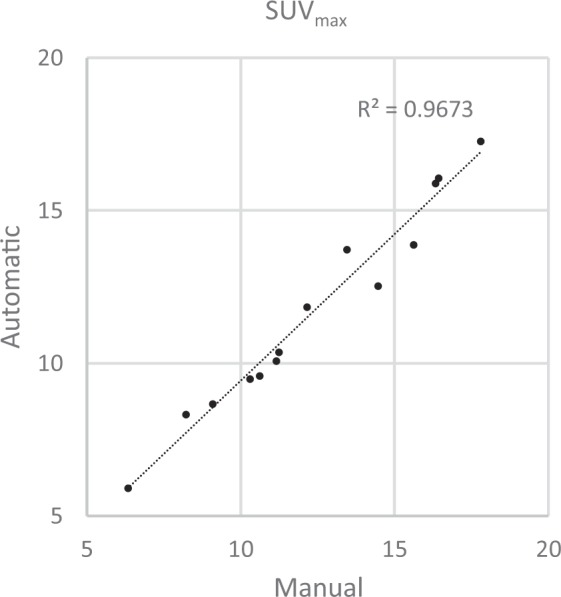
Correlation for SUV_max_ for corresponding tumours detected automatically and outlined manually. The automatic method failed to separate close-lying manually outlined lesions on five occasions. The measured characteristics for these manual lesions were combined, meaning characteristics from 14 corresponding tumours in two subjects are plotted.

After applying the lesion size and FDG uptake restrictions to the manual segmentation, 20 lesions remained (18 and 2 for the female and male subject, respectively). 19 of these lesions were detected by the automatic method. The automatic method however failed to separate lesions on five occasions, for which two manually outlined close-lying lesions were detected as one. Good agreement was obtained when comparing SUV_max_ for corresponding manual and automatically defined lesions (Fig. 6).

**Figure 7:**
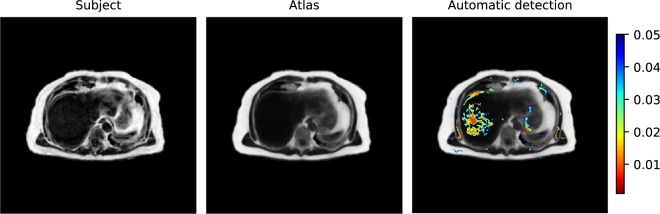
Fat fraction anomaly detection task performed for a male subject with an elevated liver fat infiltration. Representative transaxial slices of the single subject fat fraction data, atlas fat fraction data and automatically detected fat infiltration. Detected fat infiltration is displayed by showing the probability map (p < 0.05) overlaid on the atlas fat fraction image. All images are in reference space.

Transaxial slices of the atlas and test subject fat fraction data, together with the results of the liver fat detection task, are shown in Fig. 7. The detection task produced a probability map with p-values ranging from 0 to 0.05 which is overlayed on the atlas fat fraction image. The test detected abnormal fat infiltration in the liver.

## Discussion

This study aimed to develop and apply a multimodality whole-body atlas of functional FDG PET and anatomical fat-water MR data. Using whole-body image registration, an atlas containing quantitative parametric maps of fat and water fraction MR, local tissue volume and FDG PET was created, with separate entries for male and female subjects. This study expands on a previously published study in which the registration method was described in detail for fat-water MR data acquired on a 1.5 T MR scanner and a whole-body atlas was given as an example usage area^7^. In the current study, an improved registration method was utilised^19^ and it was shown that the concept of a whole-body atlas can be moved to a 3.0 T PET/MR scanner. Inherently registered PET and MR data can in this way be incorporated into the atlas. Furthermore, quantitative measurements on fat content, SUV and tissue volume were in this study performed to assess the adequacy of the content of the atlas. Specific aspects of incorporating PET into the atlas have also been described in this study, including intensity normalisation and bias assessment. As proof of concept, voxel-wise statistical comparisons were performed between the atlas and (i) two subjects with suspected malignant disease and (ii) one subject with elevated liver fat infiltration. Automated whole-body detection tasks are hence possible using the atlas.

The image registration procedure proved robust for the majority of subjects included in the atlas. Problems were however encountered, with three subjects excluded due to poor image registration results in the abdominal area. In general, this is a challenging region for the registration method due to the large physiological variability seen in this part of the body. It was also noted that standardised subject positioning is paramount for good registration results, seen both as registration failures occurred for two subjects and in the increased variability for the feet in the atlas. The registration accuracy could potentially also be affected by the choice of reference subject, ideally chosen to not bias the final atlas. In this study male and female reference subjects were chosen based on BMI and visual assessment of image quality. An alternative method could be to use group wise registration^24^, as such avoiding the uncertainties associated with choosing a single reference subject.

To create an atlas containing normal physiological values, subjects that exhibited metal artefacts in the MR images were excluded (n = 11). This was necessary as the artefacts both impact the image registration accuracy and also affect the MR intensity values. The latter also has an effect on the attenuation correction values used in PET image reconstruction and subsequently the PET intensity values. MR images containing metal implant artefacts can however be registered to and statistically compared with the atlas. Depending on the severity of the metal artefact, this could however affect the registration accuracy locally or globally. In addition, when performing voxel-wise statistical analysis, the area of metal artefact will most likely show a statistically significant change compared to the atlas. Further studies on the impact of metal artefacts on both the registration accuracy and statistical analysis are needed to fully understand the effect on automated analysis.

Manual tissue segmentations were performed in reference and native space and average fat fraction, organ volume and SUV_mean_ were measured for atlas subjects (Fig. 4). A healthy liver fat fraction of <5.56% is commonly used^25^, which tallies well with the measurements performed in this study (mean 3.0% for males and 2.4% for females). The amount of muscle fat infiltration is also comparable to previous studies, but published numbers vary due to both sequence parameters^26^ and choice of muscle group^27^. The mean organ volumes obtained for the atlas subjects overlap with previous publications^28–30^, but there is a tendency for smaller volumes measured in particular for the female cohort. Differences in the imaging protocols and segmentation methods have been shown to affect organ volumetry^31^, which could be an explanation to the discrepancy. As this study utilises the Jacobian determinant data to calculate organ volume, the registration accuracy also affects the measurements. A further reason for the somewhat smaller organ volumes could be differences in breathing instructions^30^. In this study, shallow breathing was utilised during image acquisition. An organ size difference between genders was observed, with the male cohort exhibiting larger mean lungs, liver and spleen volumes compared to the female cohort. This can to some extent be explained by the larger body size of the male cohort (Table 2). SUV_mean_ corresponded well with previously published data^32^. It should be noted, however, that the FDG uptake time in this study was close to 2 h. No quantitative bias was seen for the majority of tissues (cerebellum, lungs, aorta, liver, spleen and muscle) when comparing measurements performed in native and reference space. However, gluteal adipose tissue measurements performed in the atlas proved significantly different compared to measurements in reference space (p < 0.05). A likely explanation for this is that an abnormal stretching of fat is occasionally obtained in the registration process, when the subject data being registered includes an area of very low fat volume and the corresponding area in the reference person includes a higher fat volume.

The automated tumour detection showed a strong correlation for SUV_max_ compared with manual outlining. It was however seen that SUV_max_ does change when measured in the registered data set (Fig. 6). To prevent this, the atlas could instead be deformed to native space and measurements performed on the original patient data. Another option which should be addressed in the future is to scale the image intensity values to preserve the total counts in the image by using the Jacobian determinant data, often referred to as modulation. In the current implementation this is not performed, and instead the count concentration is preserved. Usage of this feature is most likely application-dependent as has been shown in brain imaging studies^33,34^. Future work also includes comparing the automated method with semi-automatic segmentation methods commonly used for PET imaging data^35^. A problem that was noted with the anomaly detection, that also needs addressing, is that normal physiological variations can be detected as non-normal. This was seen as false-positive lesions for e.g. physiological FDG uptake in the brain, bladder, heart and intestines. For the anomaly detection on fat infiltration, the probability map highlighted the liver as significantly different compared to the atlas.

As a proof of concept, the utility of the fat-water MR and FDG parts of the atlas was included in this study. However, the local tissue volume component of the atlas can be used in the same manner and has potential usage areas in e.g. conditions affecting organ size. Systemic diseases benefit the most from the whole-body approach that Imiomics provides. The voxel-by-voxel correspondence that is achieved makes group-wise analysis and correlation studies potential usage areas, as well as longitudinal studies of the same subject scanned over time. Furthermore, it is possible to create organ and tissue segmentations in the atlas, which after registration can be applied to individual subjects. This could be used as an aid to speed up the usually time-consuming manual outlining procedure.

This study has some limitations. Conservative inclusion criteria were used when creating the atlas, with 51 subjects excluded due to suspected pathology, poor image quality or protocol non-adherence. This is necessary in order to create a healthy control atlas, but it has the consequence of reducing the power of the atlas. A larger cohort would be needed to counter this issue. The atlas contains a limited age span (51–76 years) and BMI range (19.2–31.4 kg/m^2^). Hence, the atlas cannot be used for paediatric cases and care should be taken for obese and younger adults. For cancer applications, however, the age span included is highly relevant. An inherent limitation of the data acquisition is the difference in acquisition time between the PET and MR data, in general giving rise to more motion artefacts in the PET data and as such spatial differences between the PET and MR data sets, in particular adjacent to moving organs. Further investigations and improvements are needed with regards to optimising the PET intensity normalisation (e.g. choice of reference region) and the statistical methods used (e.g. correction for multiple statistical tests).

To conclude, a fat and water fraction MR, local tissue volume and FDG PET normal atlas was created using data acquired on a hybrid PET/MR scanner and a newly introduced image registration method. As a proof of concept, two anomaly detection tasks were performed using the atlas. The first task successfully detected non-normal FDG uptake for two subjects with suspected tumour disease. The second task automatically found abnormalities in liver fat infiltration. This proof of concept has thus demonstrated that automated whole-body voxel-wise analysis is possible for these multiparametric datasets.

## Supplementary information

Supplementary Video S1

Supplementary Video S2

## Data Availability

The datasets generated and analysed during the current study are available from the corresponding author on reasonable request.
